# CCL18通过ANXA2促进肺腺癌的侵袭

**DOI:** 10.3779/j.issn.1009-3419.2021.103.07

**Published:** 2021-07-20

**Authors:** 梓坤 邓, 钦佩 肖, 远航 郑, 瑞军 冯, 智梅 盛, 宝刚 张

**Affiliations:** 261042 潍坊，潍坊医学院临床病理系；潍坊医学院附属医院病理科 Department of Clinical Pathology; Department of Pathology, Weifang Medical University, Weifang 261042, China

**Keywords:** ANXA2, CCL18, 肺肿瘤, 侵袭, ANXA2, CCL18, Lung neoplasms, Invasion

## Abstract

**背景与目的:**

ANXA2在癌症进展中起着非常重要的作用，趋化因子18（chemokine ligand 18, CCL18）与肺腺癌（lung adenocarcinoma, LUAD）的侵袭、迁移、转移及预后不良有关。本研究旨在探究CCL18是否通过ANXA2促进LUAD侵袭以及其在LUAD侵袭中的作用和分子机制。

**方法:**

Western blot检测LUAD组织与癌周正常组织中ANXA2表达量，并检测转染效率及ANXA2作为上游调节剂在AKT/cofilin信号通路中的作用。细胞趋化实验、Transwell侵袭实验等实验探讨ANXA2对LUAD的作用机理。F-actin聚合实验和Western blot检测转染SiRNA的A549细胞侵袭是否与F-actin相关。

**结果:**

与相邻非肿瘤组织相比，癌组织中ANXA2的蛋白表达水平升高（*P* < 0.05）。敲低ANXA2组的LUAD细胞中CCL18诱导的趋化运动能力和侵袭能力下降（*P* < 0.05）。与对照组相比，敲低ANXA2的LUAD细胞的F-actin聚合明显降低，而敲低ANXA2的LUAD细胞中AKT在Ser473和Thr308位点的磷酸化和cofilin、LIMK的磷酸化水平均降低（*P* < 0.05）。

**结论:**

敲低ANXA2可以通过降低AKT及下游通路磷酸化，进而降低CCL18对LUAD细胞的侵袭性的影响。

## Abstract (en)

**Background and objective:**

ANXA2 plays a very important role in cancer progression. chemokine ligand 18 (CCL18) is associated with the invasion, migration, metastasis and poor prognosis of lung adenocarcinoma (LUAD). In this study, we aimed to explore whether CCL18 promotes LUAD invasion through ANXA2, and its role and molecular mechanism in LUAD invasion.

**Methods:**

Western blot was used to detect ANXA2 expression in LUAD tissues and adjacent non-tumor tissues, the transfection efficiency of SiANXA2#2 in cells and the role of ANXA2 as an upstream regulator in the AKT/cofilin signaling pathway. *In vitro* cytological experiments such as chemotaxis experiment and transwell invasion test was used to explore the mechanism of ANXA2 on LUAD metastasis. F-actin polymerization experiment and Western blot were used to detect whether invasion ability alteration of SiANXA2#2 A549 cells are related to F-actin.

**Results:**

Western blot analysis showed that compared with adjacent non-tumor tissues, the protein expression level of ANXA2 in cancer tissues increased (*P* < 0.05). In the chemotaxis experiment and invasion experiment, the chemotaxis and invasion ability induced by CCL18 decreased when ANXA2 knockdowned (*P* < 0.05). Compared with the control group, F-actin polymerization was significantly lower in ANXA2 knockdown group, while phosphorylation of AKT at Ser473 and Thr308 and phosphorylation of Cofilin and LIMK were reduced in ANXA2 knockdown group (*P* < 0.05).

**Conclusion:**

ANXA2 knockdown can reduce the invasive effect of CCL18 on LUAD cells by reducing phosphorylation of AKT and downstream pathways.

肺癌是造成我国患者死亡的主要原因之一，其中，肺腺癌（lung adenocarcinoma, LUAD）患者的数量近年来增长迅速，已经在肺癌发病率中占据第一位^[1, 2]^，严重威胁患者的身体健康。LUAD侵袭和转移是引起患者死亡的主要原因^[3, 4]^，大多数患者患有非小细胞肺癌（non-small cell lung cancer, NSCLC），且为晚期。组织学上可分为LUAD和鳞状细胞癌，LUAD是最常见的亚型，占所有NSCLC的50%以上^[5]^。由于LUAD患病率逐年增高，因此探究LUAD侵袭和转移分子机制是提高LUAD患者生存质量的关键。前期研究^[6, 7]^表明，膜联蛋白A2（annexin II, ANXA2）在多种癌症的发生发展中起到关键作用，ANXA2的磷酸化程度升高被认为促进癌症的转移与侵袭。前期试验已经证实，在LUAD患者癌组织中，ANXA2的表达水平显著升高，但其表达水平升高机制鲜有相关报道。趋化因子18（chemokine ligand 18, CCL18）是来源于TAMs分泌的一种癌症细胞因子^[7, 8]^，相关研究表明，ANXA2受控于CCL18与细胞表面受体Nir1相结合^[9-11]^，CCL18与受体结合后通过多种途径影响癌症的发展，其中最为显著的是发生侵袭和转移^[12]^。肌动蛋白是一类形成微丝的球状多功能蛋白质，F-肌动蛋白（F-actin）聚合成为G-肌动蛋白是细胞运动的基础，即当癌细胞运动能力发生变化时，F-actin聚合会随之变化^[13]^并受上游AKT通路影响^[14]^，本研究运用转染技术，降低ANXA2表达后，用蛋白质印迹检测ANXA2蛋白表达水平。通过体外实验检测转染ANXA2-siRNA和ANXA2过表达载体后A549细胞侵袭能力的改变，并采用F-actin聚合实验检测F-actin的变化，进而探究CCL18通过何种机制影响ANXA2及癌症的侵袭及转移。

## 1 材料与方法

### 1.1 患者和标本

2016年1月1日-2019年12月31日潍坊医学院附属医院病理科获得的患者组织标本。这些组织标本由14例浸润性LUAD及其癌旁组织的样本组成。浸润性LUAD与其相应的癌旁组织之间的距离 > 5 cm。患者的平均年龄为47.3岁（范围：35岁-76岁）。

### 1.2 材料

LUAD细胞株A549购自美国ATCC细胞库。细胞裂解液、胰蛋白酶、蛋白Marker，质粒中量抽提试剂盒，反转录试剂盒均购自TaKara公司。RPMI-1640培养基以及胎牛血清（fetal bovine serum, FBS）购自美国Hyclone公司。抗ANXA2抗体、抗p-ANXA2抗体、抗AKT抗体、抗p-AKT-Ser473抗体、抗p-AKT-Thr308抗体、抗cofilin抗体、抗p-cofilin抗体、抗LIMK抗体、抗p-LIMK抗体以及抗β-actin抗体均购自Cell Signaling公司。Transwell小室购自BD公司。Lipofectamine 2000 Reagent购自Invitrogen公司。

### 1.3 细胞培养

A549细胞培养于含10%胎牛血清、100 µg/mL青霉素和链霉素的RPMI-1640培养基中。于5%CO_2_、37 ℃细胞孵育箱中传代培养。待细胞生长处于对数期进行铺板，尽量铺匀，控制好细胞数量，6 h后观察细胞生长密度，24 h后进行瞬时转染。

### 1.4 细胞转染

按照说明书，用慢病毒载体进行转染。ANXA2 siRNA的序列5′-GGTCTGAATTCAAGAGAAA-3′和5′-GCCAAAGAAATGAACATTC-3′。将ANXA2-SiRNA转染细胞标记为SiANXA2/A549，ANXA2-SiRNA以及ANXA2过表达载体（Vector-ANXA2）共转染细胞标记为SiANXA2+ANXA2/A549，将加干扰SiRNA作为空白对照组（Scr/A549）。将A549细胞计数并铺板在6孔板内培养24 h。待细胞生长密度约70%时进行转染，并标记组别。并将病毒悬液加入到6孔板内，孵育48 h。用含有600 ng/mL潮霉素B的培养基选择稳定转染的细胞。

### 1.5 Western blot实验

将全细胞蛋白质提取物在6孔板中用裂解缓冲液裂解并匀浆，并在12, 000 rpm、4 ℃下离心15 min，使用BCA法测定蛋白质浓度。在电泳转膜后，与特异性抗体（一抗浓度为1:1, 000）一起4 ℃过夜孵育。随后将含有免疫复合物的纤维素膜与荧光素偶联的二抗一起孵育（二抗浓度为1:5, 000），然后通过Odyssey荧光扫描仪进行检测。

### 1.6 Transwell侵袭实验

取Matrigel胶40 μL混合液加入Transwell上室，将小室放入24孔板内，37 ℃放置3 h。将细胞消化离心后制备细胞（4×10^5^个/mL）悬液。在下室加入含500 μL 10%FBS的培养基，上室加入无血清细胞悬液，放在培养箱内孵育24 h。将小室取出，PBS清洗，用棉签轻轻擦掉上室未穿过的细胞。将上室用4%多聚甲醛固定并晾干，然后将上室置于24孔板，用吉姆萨工作液染色，将小室置于显微镜下观察并拍照，计算细胞数。

### 1.7 细胞趋化实验

用胰酶消化细胞，终止消化后离心弃培养基，用PBS洗3遍，用含1%牛血清白蛋白的无血清RPMI重悬细胞，调整细胞浓度为5×10^5^个/ mL。将10 ng/mL CCL18作为化学引诱剂加入下室，上室加200 µL细胞悬液，在培养箱孵育24 h后，甲醛固定30 min。用0.1%吉姆萨染色20 min，用棉签擦掉上室未迁移的细胞。用显微镜以×200的倍数随机观察3个视野。

### 1.8 F-actin聚合实验

将细胞铺在6孔板内常规培养，将细胞放在不含血清的培养基中37 ℃、5%CO_2_培养箱内，细胞饥饿3 h。用10 ng/mL的CCL18分别刺激15 s和30 s、1 min、2 min和3 min后固定，用预冷的PBS冲洗细胞，终止CCL18的刺激。用4%PFA固定细胞10 min。F-buffer（10 mmol/L HEPES, 20 mmol/L KH_2_PO_4_, 5 mmol/L EGTA, 2 mmol/L MgCl_2_, Dulbecco's PBS, pH 6.8）冲洗3次，每次5 min。孔内加1 mL 0.5%Tritox-100，静置20 min。F-buffer冲洗3次，每次5 min。孔内加荧光鬼笔环肽，避光孵育1 h。每孔加入900 µL纯甲醇，4 ℃孵育60 min。1, 500 rpm，离心5 min，将上清液移入96孔板内，每孔加3个复孔。使用酶标仪读取数值，荧光的激发波长488 nm，散发波长530 nm。不同时间的F-肌动蛋白相对聚合量计算公式为：刺激时间F-actin值/未刺激F-actin值=刺激时间荧光值。每组实验至少重复3遍。取平均值作为实验结果。

### 1.9 统计学方法

所有实验数据运用SPSS 16.0分析。计量资料之间的比较采用独立样本*t*检验。多组间的比较采用单因素方差分析。所有数据采用均数±标准差（Mean±SD）的形式表示。*P* < 0.05被认为差异有统计学意义。

## 2 结果

### 2.1 ANXA2在LUAD中的表达及其磷酸化程度与CCL18的关系

通过Western blot分析浸润性导管癌组织和邻近的非肿瘤组织的ANXA2表达水平，结果显示，在4例具有代表性的患者样本中，与相邻非肿瘤组织相比，LUAD癌组织中ANXA2的蛋白表达水平明显增高（*P* < 0.05，图 1A）。随后我们统计了ANXA2在所有患者样本癌组织和相邻非肿瘤组织中的蛋白表达，得到了相同的结论（*P* < 0.05，图 1B）。将Scr/A549细胞作为对照组。通过Western blot检测在有或没有CCL18刺激的情况下，Scr/A549和SiNir1/A549细胞中ANXA2的蛋白和磷酸化水平，结果显示，Nir1的表达影响CCL18诱导的ANXA2磷酸化。即CCL18与Nir1的结合促进了LUAD细胞中ANXA2的磷酸化水平，但没有促进ANXA2的蛋白表达水平（*P* < 0.05，图 1C）。

**图 1:**
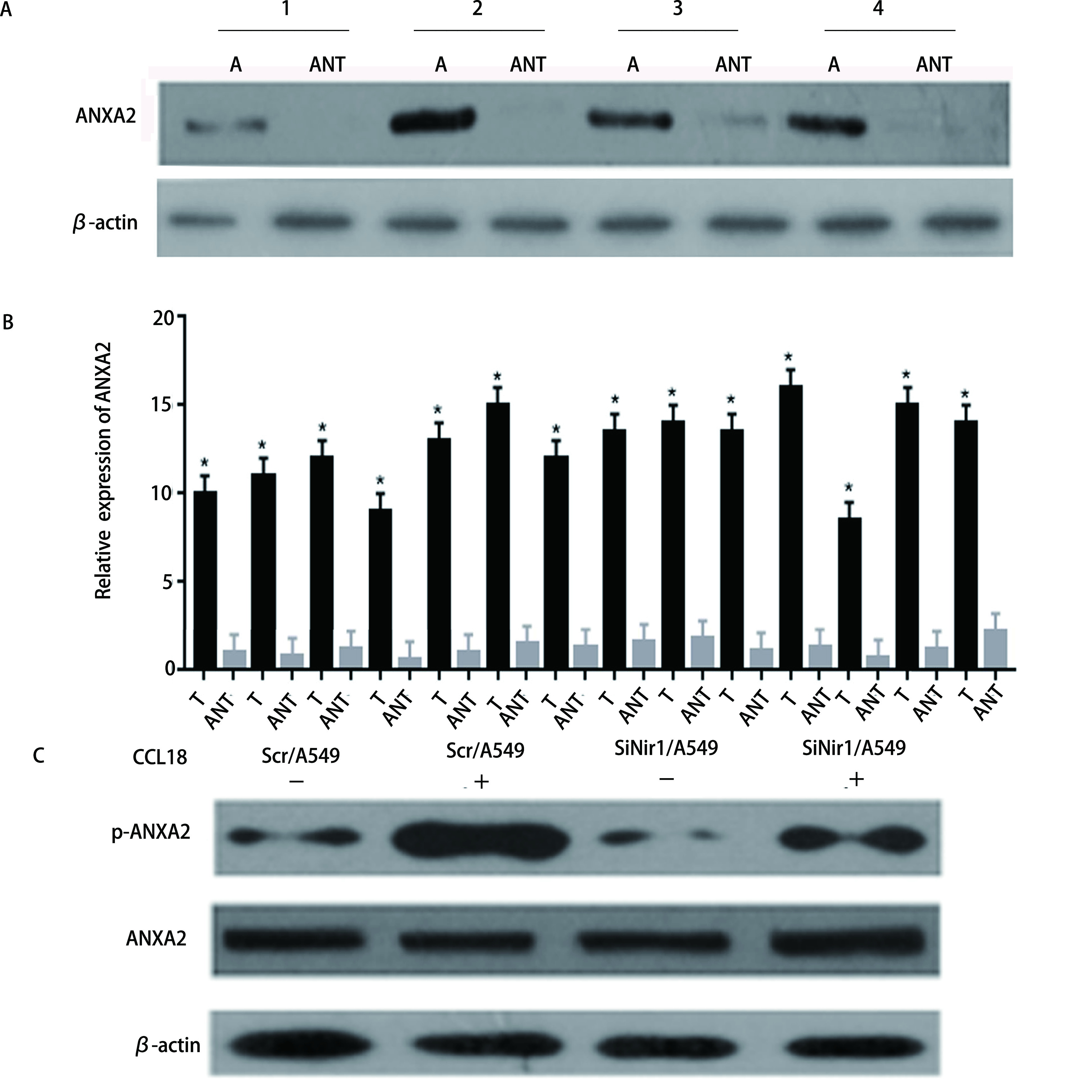
ANXA2在肺腺癌细胞中过表达，其磷酸化受到CCL18的调控。A：ANXA2蛋白在配对的肺腺癌组织和邻近正常组织中的表达情况，每对均来自同一患者；B：柱状图统计ANXA2蛋白在配对的肺腺癌组织和邻近正常组织中的表达情况；C：通过蛋白质印迹分析技术检测在有或没有10 ng/mL的CCL18刺激下，Scr/A549和SiNir1/A549细胞中ANXA2蛋白的表达水平。 ANXA2 is overexpressed in lung adenocarcinoma cells, and its phosphorylation is regulated by CCL18. A: The expression of ANXA2 protein in paired lung adenocarcinoma tissue and adjacent normal tissue, each pair comes from the same patient; B: The expression of ANXA2 protein in paired lung adenocarcinoma tissue and adjacent normal tissue was analyzed by histogram; C: The expression level of ANXA2 protein in Scr/A549 and SiNir1/A549 cells was detected by Western blot analysis with or without CCL18 stimulation of 10 ng/mL. T: lung adenocarcinoma tissue; ANT: adjacent normal tissue.

### 2.2 ANXA2的降低可抑制LUAD细胞的趋化运动能力以及侵袭能力

使用siRNA降低A549中ANXA2蛋白的水平，我们通过Western blot分析验证在稳定转染的A549细胞中ANXA2蛋白表达水平出现了下降（*P* < 0.05，图 2A）。通过细胞趋化实验发现，在降低了ANXA2蛋白表达之后，CCL18诱导的趋化运动出现了显著的下降，表明ANXA2促进了LUAD细胞中CCL18诱导的趋化运动能力（*P* < 0.05，图 2B）。侵袭实验表明，无论培养基中是否加入CCL18，SiANXA2#2/A549组穿过人工基质胶的细胞数量明显少于Scr/A549组（*P* < 0.05，图 2C），这表明ANXA2的降低抑制了CCL18诱导的LUAD细胞粘附、迁移和侵袭能力。

**图 2:**
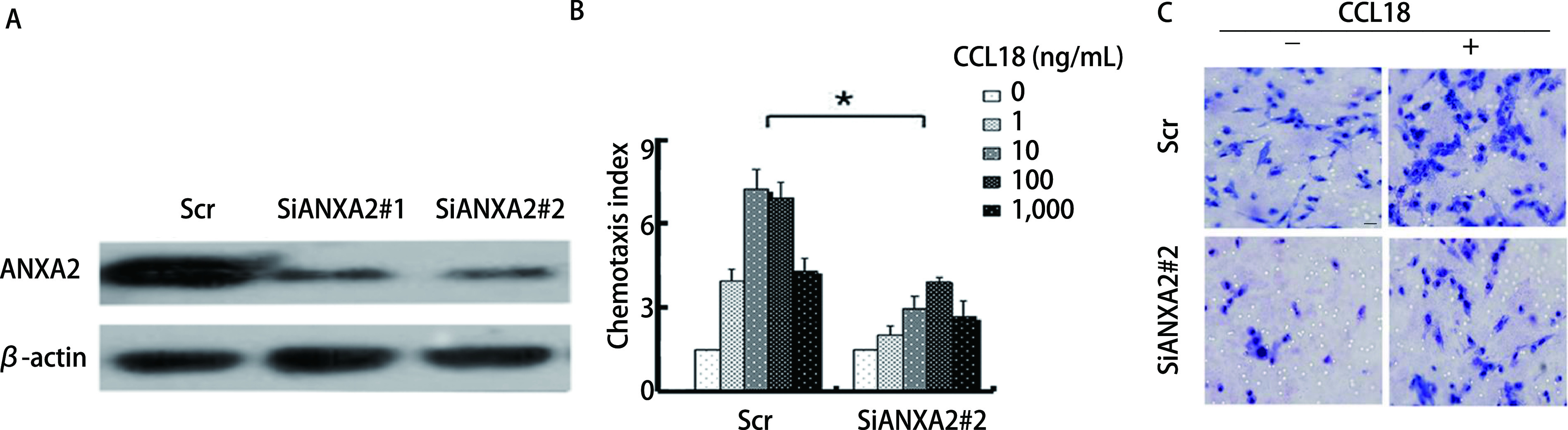
ANXA2促进CCL18诱导的肺腺癌细胞趋化运动能力。A：在以Scrabble siRNA作为对照（Scr/A549）和两组稳定的siRNA（SiANXA2#1/A549和SiANXA2#2/A549）转染A549细胞后检测ANXA2蛋白的表达水平；B：用CCL18刺激Scr/A549和SiANXA2#2/A549细胞，进行趋化反应的比较。*β*-actin作为阴性对照。每独立实验至少重复3次, *P* < 0.05，差异有统计学意义；C：在加入或不加入10 ng/mL CCL18刺激的情况下，分析Scr/A549和SiANXA2#2/A549细胞的侵袭能力，*P* < 0.05。以×400放大倍数捕获图像，比例尺：20 μm。 ANXA2 promotes the chemotaxis of lung adenocarcinoma cells induced by CCL18. A: Two stable siRNAs (SiANXA2#1 and SiANXA2#2) were transfected into A549 cells respectively, scramble siRNA as a control (Scr). The ANXA2 protein expression level was detected by Western blot analysis. *β*-actin was used as a negative control; B: Comparison of chemotactic response with rCCL18 stimulation in Scr/A549 and SiANXA2#2/A549 cells. Each independent experiment was repeated at least three times, *P* < 0.05, the difference was statistically significant; C: Analyze the invasion ability of Scr/A549 and SiANXA2#2/A549 cells with or without rCCL18 stimulation of 10 ng/mL, *P* < 0.05. Capture images at ×400 magnification. Scale bar: 20 μm.

### 2.3 ANXA2的降低可通过AKT/cofilin信号通路抑制LUAD中CCL18诱导的F-actin聚合

为了证明ANXA2的下调可以通过抑制F-actin聚合来抑制CCL18诱导的LUAD细胞趋化运动能力这一假设。F-actin聚合实验结果表明，CCL18在Scr/A549细胞中在15 s、30 s、1 min、2 min和3 min时引起瞬时肌动蛋白聚合，在SiANXA2#2/A549细胞中，肌动蛋白的聚合反应显著降低（*P* < 0.05，图 3A），即ANXA2通过调节CCL18诱导的F-actin聚合进而调节LUAD运动和侵袭。用10 ng/mL CCL18刺激细胞3 min后，通过Western blot分析实验检测转录因子的核表达，以验证参与F-actin的下游分子，这些分子在细胞中受ANXA2的调控。与Scr/A549细胞相比，SiANXA2#2/A549细胞中Ser473和Thr308位点的AKT的磷酸化水平均降低（*P* < 0.05，图 3B）。Western blot结果表明，与Scr/A549组相比，SiANXA2#2/A549细胞中cofilin及LIMK磷酸化水平降低（*P* < 0.05，图 3C）。这些结果表明，AKT/cofilin信号通路对于ANXA2促进LUAD细胞中CCL18诱导的趋化运动及侵袭能力至关重要。

**图 3:**
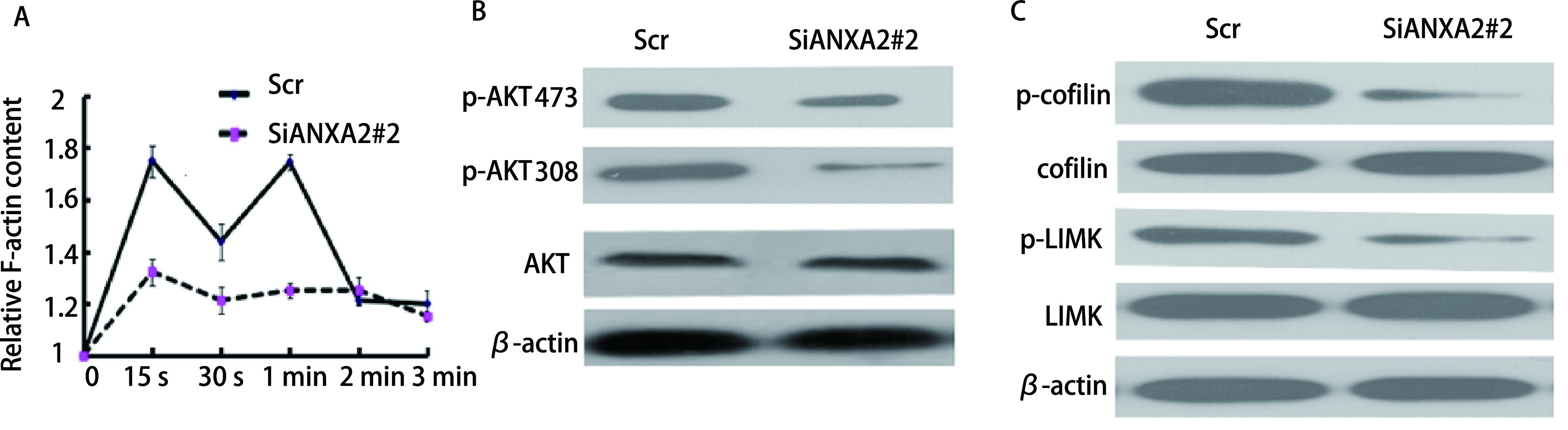
ANXA2的降低通过AKT/cofilin通路抑制肺腺癌细胞中CCL18诱导的F-肌动蛋白聚合。A：在不同时长的10 ng/mL CCL18的刺激下Scr/A549以及和SiANXA2#2/A549细胞中相对F-actin含量；B：在CCL18刺激下，分析Scr/A549、SiANXA2#2/A549中p-AKT（Ser473）、p-AKT（Thr308）的表达水平，实验中使用AKT和*β*-actin用作阴性对照；C：分析在CCL18的刺激下，Scr/A549、SiANXA2#2/A549中cofilin、LIMK的表达水平，实验中使用cofilin、LIMK和*β*-actin用作阴性对照。 The reduction of ANXA2 inhibits CCL18-induced F-actin polymerization in lung adenocarcinoma cells through the AKT/cofilin pathway. A: Relative F-actin content in Scr/A549 and SiANXA2#2/A549 cells stimulated by 10 ng/mL CCL18 at different times; B: Under the stimulation of CCL18, the expression levels of p-AKT (Ser473) and p-AKT (Thr308) in Scr/A549 and SiANXA2#2/A549 were analyzed by Western blot analysis. AKT and *β*-actin were used as negative contrast in the experiment; C: Analyze the expression levels of cofilin and LIMK in Scr/A549, SiANXA2#2/A549 under the stimulation of CCL18 by Western blot analysis. In the experiment, cofilin, LIMK and *β*-actin were used as negative controls.

### 2.4 过表达ANXA2可促进LUAD细胞侵袭能力和LUAD中CCL18诱导的F-actin聚合

为了证明ANXA2对LUAD细胞功能的影响，我们进行了挽救实验，使用SiRNA降低A549中ANXA2蛋白的水平，过表达ANXA2载体（Vector-ANXA2）恢复ANXA2蛋白的水平，我们通过Western blot分析验证，结果表明A549细胞稳定转染（图 4A）。侵袭实验表明，无论培养基中是否加入CCL18，SiANXA2#2+ANXA2/A549组穿过人工基质胶的细胞数量明显多于SiANXA2#2/A549组（*P* < 0.05，图 4B）。F-actin聚合实验结果表明，与SiANXA2#2/A549组相比，过表达ANXA2后，肌动蛋白的聚合反应显著升高（*P* < 0.05，图 4C）。这些结果表明，ANXA2过表达可促进LUAD细胞侵袭能力和LUAD中CCL18诱导的F-actin聚合。

**图 4:**
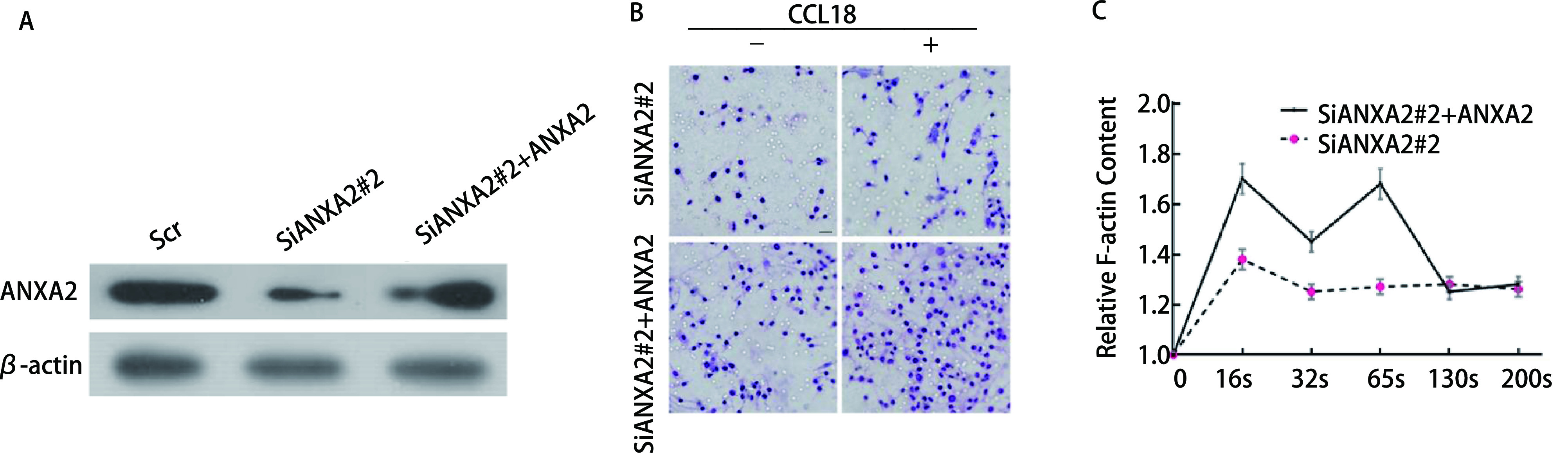
过表达ANXA2可促进肺腺癌细胞侵袭能力和肺腺癌中CCL18诱导的F-actin聚合。A：Western blot检测SiANXA2#2和SiANXA2#2+ANXA2转染效率；B：在加入或不加入10 ng/mL CCL18刺激的情况下，分析SiANXA2#2/A549和SiANXA2#2+ANXA2/A549细胞的侵袭能力，*P* < 0.05。以×400放大倍数捕获图像，比例尺：20 μm；C：在不同时长的10 ng/mL CCL18的刺激下SiANXA2#2/A549以及和SiANXA2#2+ANXA2/A549细胞中相对F-肌动蛋白含量。 Overexpression of ANXA2 promotes invasion of lung adenocarcinoma cells and CCL18-induced F-actin aggregation in lung adenocarcinoma cells. A: The transfection efficiency of SiANXA2#2 and SiANXA2#2+ANXA2 was detected by Western blot; B: Analyze the invasion ability of SiANXA2#2/A549 and SiANXA2#2+ANXA2/A549 cells with or without rCCL18 stimulation of 10 ng/mL. *P* < 0.05. Capture images at ×400 magnification. Scale bar: 20 μm; C: Relative F-actin content in SiANXA2#2/A549 and SiANXA2#2+ANXA2/A549 cells stimulated by 10 ng/mL CCL18 at different times.

## 3 讨论

尽管在肺癌筛查及外科医学、放射肿瘤学治疗方面的不断进步的前提下，LUAD仍然是中国乃至世界范围内最常见的癌症死亡原因^[15]^。而侵袭和转移是造成绝大多数患者死亡的重要原因^[16]^。所以探究LUAD侵袭和转移分子机制是提高LUAD患者生存质量的关键^[17]^。研究表明，ANXA2可以促进人类结直肠癌^[18]^、胃癌^[19]^和乳腺癌^[20]^等的侵袭和转移，在LUAD的侵袭转移中，同样也有促进血管生成和癌组织增殖等作用^[21]^。但在其如何受到CCL18调控，以及通过何种方式影响癌症转移及侵袭鲜有研究。

本研究中，我们使用了蛋白质印迹分析的方法对14例患者的LUAD组织进行了分析，我们从中精选了4例具有代表性的患者组织做了Western blot，结果显示，相对于相邻周围组织，癌组织中ANXA2的表达量升高。并随后做了柱状图统计ANXA2在14例患者组织中的表达，得到的结果再次证明了上述结论。相关研究^[22]^显示，其水平升高与LUAD的转移侵袭相关。在稳定转入SiNir1的A549细胞中，与对照组Scr/A549细胞相比，ANXA2的磷酸化水平发生了下降，而当没有CCL18的刺激时，ANXA2磷酸化明显降低，即ANXA2在细胞内的磷酸化受到CCL18的调控，这在过往其他癌相关文献^[23]^中也有提及。同时，Scr/A549和SiANXA2#2/A549的细胞运动学实验表明抑制ANXA2后A549细胞侵袭能力明显下降，而在恢复ANXA2的表达后，A549细胞的侵袭能力较SiANXA2#2组明显上升。在对其进行F-actin聚合实验分析结果，F-actin聚合反应参与了对癌细胞侵袭转移的影响。随后我们采用Western blot分析的实验方法，对F-actin聚合相关的上游AKT/cofilin通路进行了检测。结果显示SiANXA2#2/A549相对于对照组，p-AKT（Ser473）、p-AKT（Thr308）、cofilin和LIMK的磷酸化都发生了降低。

综上所述，ANXA2通过AKT/cofilin信号通路促进CCL18诱导的细胞运动能力增强，促使LUAD发生转移及侵袭，为CCL18成为临床筛查LUAD及靶向治疗的分子标志物奠定理论基础，提高LUAD患者的远期生存率。虽然我们做了CCL18对ANXA2相关影响的研究，但是其对LUAD发生和进展的蛋白机制并未完全阐明，还需进一步研究。
